# Full preclinical validation of the 123I-labeled anti-PSMA antibody fragment ScFvD2B for prostate cancer imaging

**DOI:** 10.18632/oncotarget.14229

**Published:** 2016-12-30

**Authors:** Barbara Frigerio, Gerben Franssen, Elena Luison, Alessandro Satta, Ettore Seregni, Marco Colombatti, Giulio Fracasso, Riccardo Valdagni, Delia Mezzanzanica, Otto Boerman, Silvana Canevari, Mariangela Figini

**Affiliations:** ^1^ Department of Experimental Oncology and Molecular Medicine, S.S. Molecular Therapies, Fondazione IRCCS Istituto Nazionale dei Tumori, Milan, Italy; ^2^ S.C. Nuclear Medicine, Fondazione IRCCS Istituto Nazionale dei Tumori, Milan, Italy; ^3^ Department of Medicine, University of Verona, Verona, Italy; ^4^ Department of Oncology and Hemato-Oncology, Universitá degli Studi di Milano, Radiation Oncology 1, Prostate Cancer Program, Fondazione IRCCS Istituto Nazionale dei Tumori, Milan, Italy; ^5^ Department of Radiology and Nuclear Medicine, Radboud University Medical Center, Nijmegen, The Netherlands

**Keywords:** scFv antibody fragment, ^123^I-radiolabeled antibody, prostate cancer, prostate-specific membrane antigen, imaging

## Abstract

**Purpose:**

In the context of prostate cancer (PCa) imaging, the aim of this study was to optimize (*in vitro*) the specificity and assess preclinically (*in vivo*) the tumor targeting properties of the ^123^I-scFvD2B antibody specific for prostate-specific membrane antigen (PSMA).

**Experimental Design:**

The ^123^I-labeling conditions of the antibody fragment scFvD2B, produced in an eukaryotic system under GMP-compliant conditions, were optimized and assessed for purity and immunoreactivity. The specificity and potency of tumor uptake were tested in three preclinical in vivo models of subcutaneously xenografted human tumors expressing different levels of PSMA (LNCaP, naturally expressing PSMA; PC3-PIP and LS174T-PSMA, transfected with PSMA) or PC3 and LS174T, as negative controls, to assess the clearance, biodistribution and imaging potential of ^123^I-scFvD2B.

**Results:**

The set conditions of production and radiolabeling yielded a reagent suitable for human delivery thanks to the purity of the formulation and the high immunoreactivity. In all preclinical models ^123^I-scFvD2B showed specific targeting only to PSMA-positive tumors with the final specific activity ranging up to 1500 MBq/mg. Despite different levels of PSMA expression, biodistribution analyses and SPECT/CT imaging demonstrated similar results and maximal signal-to-background ratios 24 hours after injection.

**Conclusions:**

Due to its in vitro and in vivo properties, ^123^I-scFvD2B could be a promising tool for the early diagnosis of PCa, and may represent a molecular imaging option to monitor disease progression and assist in the clinical management of PCa patients.

## INTRODUCTION

Prostate cancer (PCa) is the second most frequently diagnosed cancer among males worldwide [1]. Due to its insidious behaviour, early diagnosis of PCa is difficult to diagnose in the early phase of the disease and many patients present with metastatic disease at diagnosis [2]. Conventional imaging techniques such as bone scintigraphy, computed tomography (CT), ultrasound, and magnetic resonance imaging are currently used to detect primary PCa and metastatic disease; however, in order to optimize the management of PCa patients improved imaging modalities are needed. In this scenario, positron emission tomography (PET) and single photon emission computed tomography (SPECT) with emerging radiopharmaceuticals may provide accurate staging of primary disease, restaging of tumor recurrence, and detection of metastatic disease. The possibility to specifically target a radiotracer strongly enhances the efficacy of these techniques.

Among the cell-surface molecules, prostate-specific membrane antigen (PSMA), which is associated with metastatic and androgen-independent disease [3], represents a promising marker for PCa. Its expression profile and biological properties make it an attractive antigen for cancer targeting. PSMA is overexpressed in the malignant epithelial cells that define PCa [4]. ^111^In-labeled capromab pendetide (ProstaScint^®^), an anti-PSMA murine monoclonal antibody [5, 6] conjugated with a derivative of DTPA [7], has been approved for diagnostic imaging by the US Food and Drug Administration but not by the European Medicines Agency, as it has shown some limitations [8]. Many ongoing studies are using radiolabeled small peptides that bind the extracellular domain of PSMA; however although some of these peptides show good localization, high tracer uptake in the kidneys and salivary glands raised a major concern [9].

Besides PCa, previous studies have found PSMA expression in the neovasculature of a wide variety of solid tumors including breast carcinoma, gynecological and head and neck cancers, renal cell carcinoma, colorectal cancer, glioblastoma, and gastric adenocarcinoma [10, 11, 12], suggesting that this target can be useful also in other tumor types, extending the range of application of the reagent described herein.

Our monoclonal antibody D2B, obtained by conventional hybridoma technology, is directed against the extracellular domain of human PSMA [13]. The antibody, as entire IgG, F(ab′)_2_ and Fab formats radiolabeled with ^111^In, had previously been evaluated for PCa imaging in comparison with ProstaScint^®^ in preclinical models where it showed good tumor uptake, markedly better than ProstaScint^®^ [14].

In order to enhance the typically low tumor penetration and slow clearance from the background of a whole antibody molecule and improve this critical parameter for effective radioimmunotherapy, we reshaped the murine monoclonal antibody D2B into a scFv polypeptide encompassing the variable regions of the heavy and light chains joined by a short peptide linker [13]. Due of its reduced size, resulting in faster blood clearance and improved tissue penetrability [14], the scFv antibody could be an ideal candidate as a diagnostic agent [15]. Biochemical and functional characterization of scFvD2B produced in a prokaryotic system showed optimal stability and good cell-interaction features despite its monovalent binding [13]. Furthermore, we demonstrated that the use of ^131^I resulted in significantly stable tumor specificity whilst minimizing the background compared with a radiometal tracer such as ^111^In [16]. In the present study, we optimized the ScFv production procedures and radioiodination conditions with ^123^I and we assessed, in two independent laboratories, the *in vivo* tumor targeting and tumor imaging potential of ^123^I-scFvD2B in three different xenograft models of human tumors expressing different levels of PSMA. The results of this study warrant the development of ^123^I-scFvD2B for clinical practice.

## RESULTS

### Characterization of scFvD2B from eukaryotic system

**Figure 1:**
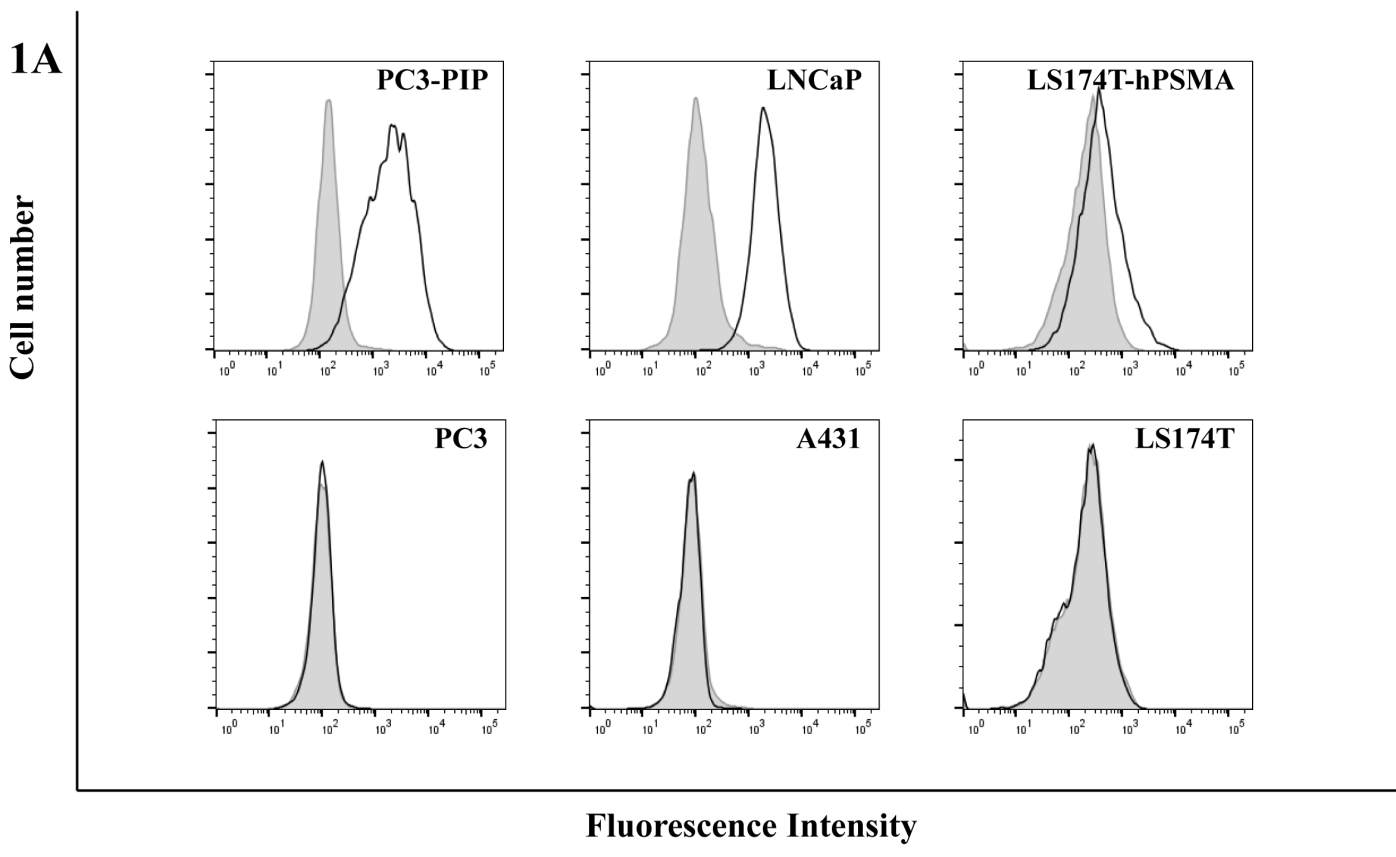

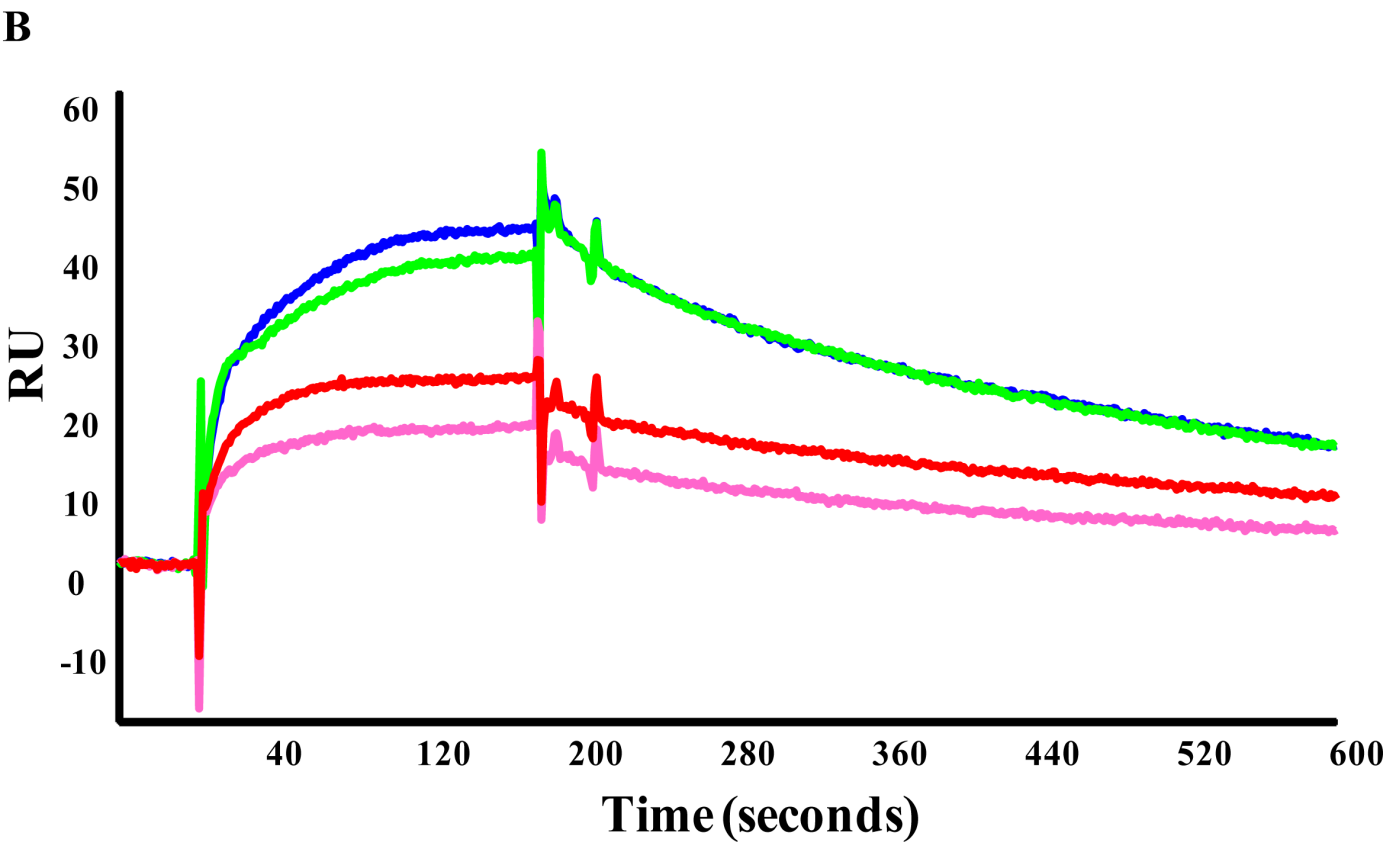
**A. Flow cytometric analysis of scFvD2B. scFvD2B** (solid line) binding on PSMA-positive cell lines (PC3-PIP, LNCaP and LS174T-PSMA) and PSMA-negative cell lines (PC3, A431, LS174T). The shift in fluorescence was assessed relatively to a negative control (gray histogram, cells incubated with biotinylated Protein L and streptavidin-PE). **B**. BIAcore analysis: scFvD2B obtained from prokaryotic system (Blue line) and from eukaryotic system (Green line) on PC3-PIP lisate; scFvD2B obtained from prokaryotic system (Red line) and from eukaryotic system (Purple line) on LNCaP lisate.

GMP scFvD2B from the eukaryotic system was produced and then purified starting from a clone in which the Myc and His tags had been removed. The product was biochemically characterized in comparison to the scFvD2B from the prokaryotic system. The theoretical MWs and pIs of the two scFvD2Bs were 26,361 and 29,095 Da and 8.80 and 7.88 pI for the eukaryotic and prokaryotic reagent, respectively. The theoretical MW and pI essentially corresponded to those experimentally determined using mass spectrometry and isolectrofocusing: 26,544 and 29,060 Da and 9.0 and 8.0 pI for the eukaryotic and prokaryotic reagent, respectively (Figure 1 Supplementary).

The binding specificity of scFvD2B from the eukaryotic system was assessed by FACS on LNCaP, PC3-PIP and LS174T-PSMA cells and PC3, A431 and LS174T cells (as negative controls), and by BIAcore on PSMA. The FACS assay demonstrated reactivity in all PSMA-positive cells expressing a wide range of PSMA levels; no binding was observed in negative controls (Figure 1A). No change in terms of binding and kinetics was seen compared with scFvD2B produced in the prokaryotic system (Figure 1B).

### ^123^I-scFvD2B radiolabeling

The radiolabeling parameters and bioactivity of ^123^I-scFvD2B from the eukaryotic system are reported in Table 1 in comparison to data obtained with ^123^I- scFvD2B from the prokaryotic system. An initial experiment, using the conditions set for prokaryotic production and ^131^I (see ref. [16]), yielded good final results with both reagents but a 50% labeling efficiency for the eukaryotic product (data not shown in the Table); this parameter was greatly improved in the following six experiments by adding cold NaI and using protein concentrations > 8 mg/mL (Table 1). These preparations were used for the *in vivo* experiments at INT reported hereafter. Since trace amounts of TRIS may be present in the final preparation even after column purification in PBS and this is not acceptable for human delivery, labeling in PBS was then tested *in vitro* at INT and *in vivo* at Radboud University. These labeling conditions resulted in quite variable labeling efficiency, ranging from 35.7% to 83.5%. Despite the different labeling efficiency, after purification by size exclusion chromotography the final radiochemical purity always exceeded 95%.

**Table 1:** Summary of radiochemical characteristics and bioactivity of scFvD2B after labeling with 123I

Characteristics of ^123^I-scFvD2B tested at	INT†			Radboud University†
**Host cells for production**	Prokaryotic	Eukaryotic	Eukaryotic	Eukaryotic
**Radiolabeling buffer**	TRIS-HCl	TRIS-HCl	PBS	PBS
**Number of experiments**	2	6	7	1
**Efficiency (%)**	96.9-100	87.7-98.5	35.7-83.5	65
**Radiochemical purity (%)**	98.5-100	98.5-100	98.4-100	>95
**Specific activity (MBq/mg)**	59.2-173.9	59.2-181.3	25.9-259	1500
**Immunoreactivity (%)** **PSMA-positive cells: PC3-PIP** **LNCaP**	71.0-74.8 64.0-73.4	77.8-93.0 77.0-89.0	85.7-90.7 72.1-79.6	ND*
**Immunoreactivity (%)** **PSMA-negative cells: PC3** **A431**	0.8 0.7-1.0	0.4-2.4 0.2-2.5	0.2-1.7 0.1-0.7	ND*

Abbreviation: INT, Istituto Nazionale dei Tumori.

† INT, preparations used for in vitro and in vivo experiments; Radboud University Medical Center, preparation used for in vivo experiments.

* Not determined in vitro, but tested in vivo on LS147T model by adding cold scFvD2B (see Figure 4).

Data reported represent the range obtained in all the experiments performed.

No change in immunoreactivity was observed when the scFvD2B from the prokaryotic system was labeled with ^123^I or ^131^I [16], while the immunoreactivity of ^123^I-scFv obtained with the eukaryotic system was significantly higher than that of ^123^I-scFv produced in the prokaryotic system (PC3-PIP, mean ± SD = 87.0±5.0 and 72.9±2.7, *P* = 0.015; LNCaP mean ± SD = 82.8±4.5 and 68.7±6.6, *P* = 0.02 for eukaryotic and prokaryotic, respectively) without any significant increase in nonspecific binding on PSMA-negative cells. The labeling buffer (PBS *versus* TRIS) did not significantly affect immunoreactivity (Table 1).

Different ratios between amounts of protein (mg) and isotope (MBq) were tested (see Figure legends) and despite a 10-fold or 50-fold increase in the specific activity (see Table 1) the final reagents retained in the PBS formulation their ability to bind PSMA *in vitro* (INT) and *in vivo* (Radboud University).

### ^123^I-scFvD2B clearance

**Figure 2:**
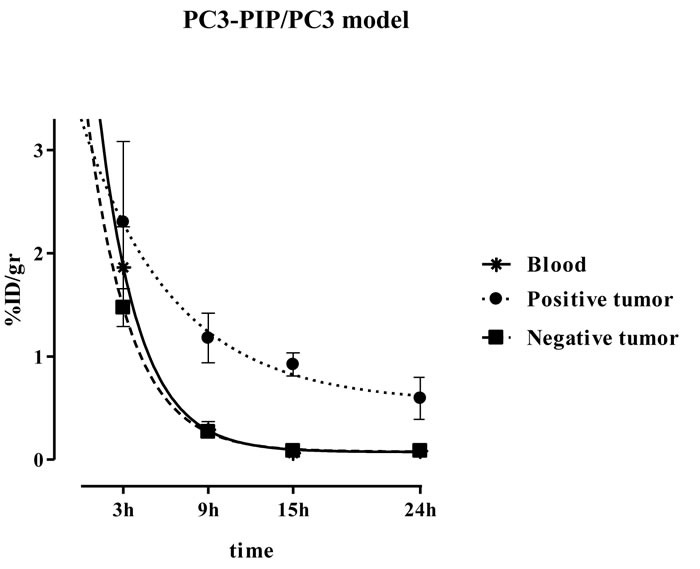
Clearance of **^123^**I-scFvD2B in tumor-bearing mice. Blood (★), PSMA-positive tumor (PC3-PIP; ·) PSMA-negative tumor ( PC3;) at different time points after injection of 7.5 MBq (100 µg; specific activity [SA] = 75 MBq/mg) ^123^I-scFvD2B (mean of 2 experiments). Values are expressed as mean %ID/g ± SD; the number of animals ranged from 3 to 14.

The different rates of clearance of ^123^I-scFvD2B from blood, PC3-PIP (PSMA-positive tumor) and PC3 (PSMA-negative tumor) are shown in Figure 2. The determined ^123^I-scFvD2B β half-life was 1.94 hours (95% CI 1.44-2.94; R^2^ = 0.96) in blood, 4.51 hours (95% CI 2.54-20.20; R^2^ = 0.78) in PC3-PIP, and 2.08 hours (95% CI 1.48-3.45; R^2^ = 0.95) in PC3. Comparison with the new formulation and the use of the ^123^I isotope yielded comparable results to the data obtained with ^131^I-scFvD2B produced in a prokaryotic system [16].

### Biodistribution studies

**Figure 3:**
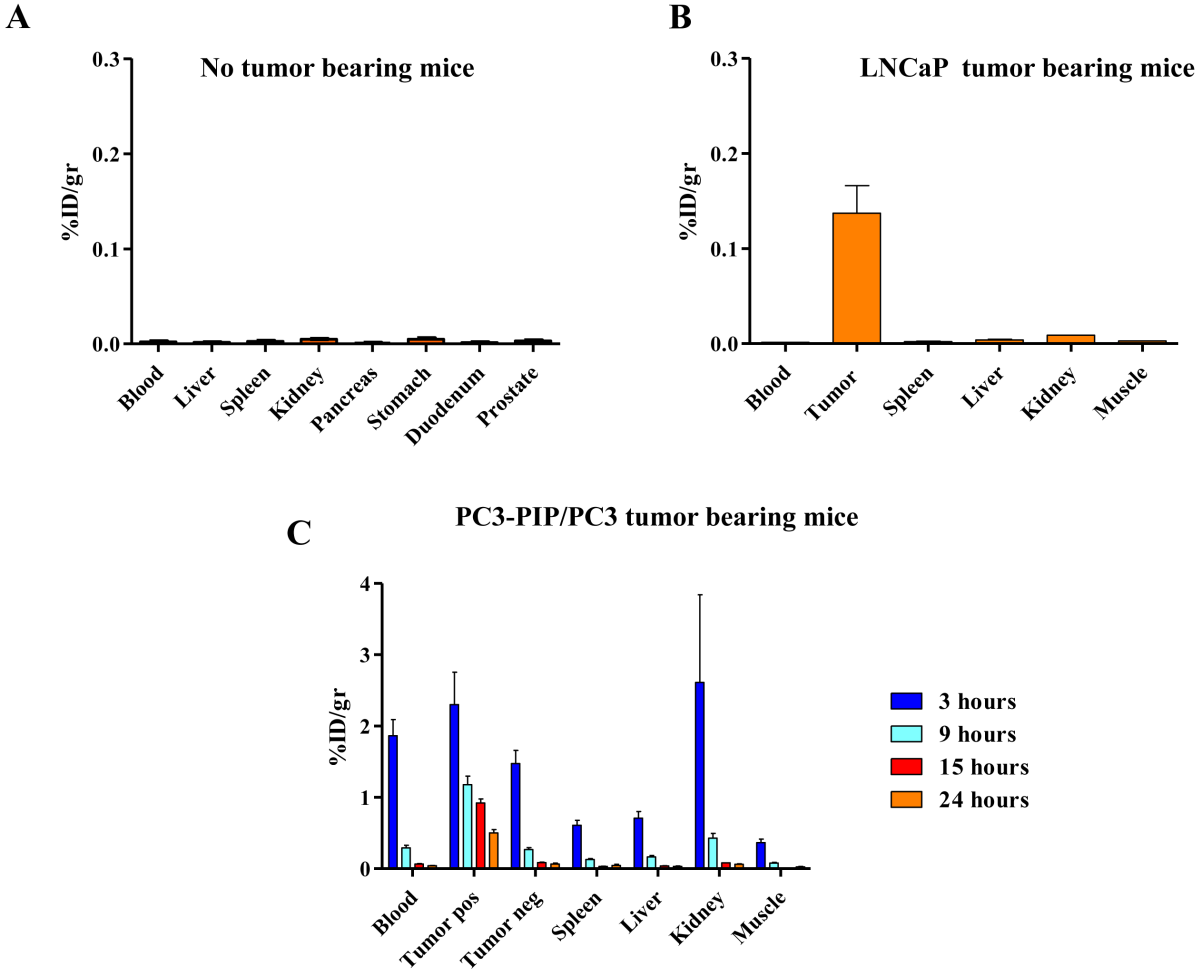
Biodistribution and localization after intravenous administration of **^123^**I-scFvD2B to athymic mice. Uptake and retention were measured in different organs as decay-adjusted percentage of the injected dose per gram of tissue (% ID/g). **A**. Biodistribution in non-tumor-bearing mice evaluated 24 hours post injection, 7.5 MBq (41 µg; SA = 181 MBq/mg) administered; error bars represent SD from the mean value of 4 mice. **B**. Biodistribution in LNCaP tumor bearing mice evaluated 24 hours post injection; 7.5 MBq (51 µg; SA = 145 MBq/mg) administered; error bars represent SD from the mean value of 2 mice. **C**. Biodistribution in PC3-PIP/PC3 tumor bearing mice evaluated at 3, 9, 15 and 24 hours post injection; 7.5 MBq (95 µg; SA = 78 MBq/mg) administered; error bars represent SD from the mean value of 3 to 6 mice.

At 24 hours after injection of ^123^I-scFvD2B, no accumulation for all examined tissues was recorded in non-tumor-bearing mice (Figure 3A), while preferential uptake of ^123^I-scFvD2B in PSMA-positive tumors (%ID/g mean ± SD = 0.14±0.03) was observed in animals engrafted with LNCaP cells (Figure 3B). The PSMA-positive tumor and kidney to blood ratios were 97.3 and 6.3, respectively.

A biodistribution study was performed in mice bearing in one flank PSMA-positive and in the controlateral flank PSMA-negative tumors (PC3-PIP and PC3 cells, respectively) at three, nine, 15 and 24 hours post injection. Overall, the data showed specific localization of the reagent; nine hours post injection the uptake in PSMA-positive tumors (%ID/g mean ± SD = 1.18±0.24) was significantly higher than in blood, kidneys and PSMA-negative tumors (%ID/g mean ± SD = 0.29±0.08, 0.43±0.13, 0.27±0.05, respectively). After 24 hours, the blood and kidneys were cleared while the uptake in the PSMA-positive tumors remained high (%ID/g mean ± SD = 0.50±0.09) (Figure 3C). The PSMA-positive tumor to blood ratios were 4.2 and 13.6 at 9 and 24 hours, respectively; the PSMA-negative tumor and kidney to blood ratios were around 1 at 24 hours.

The results were confirmed also in the experiments performed using the LS174T-PSMA/LS174T model (Figure 2 Supplementary). The PSMA-positive tumor blood ratios were 21.5 and 37.3 at 9 and 24 hours, respectively; the PSMA-negative tumor and kidney blood ratios were around 2 at 24 hours. Coinjection of 100-fold excess cold reagent reduced the uptake in PSMA-positive tumors to background levels.

### Imaging studies

**Figure 4:**
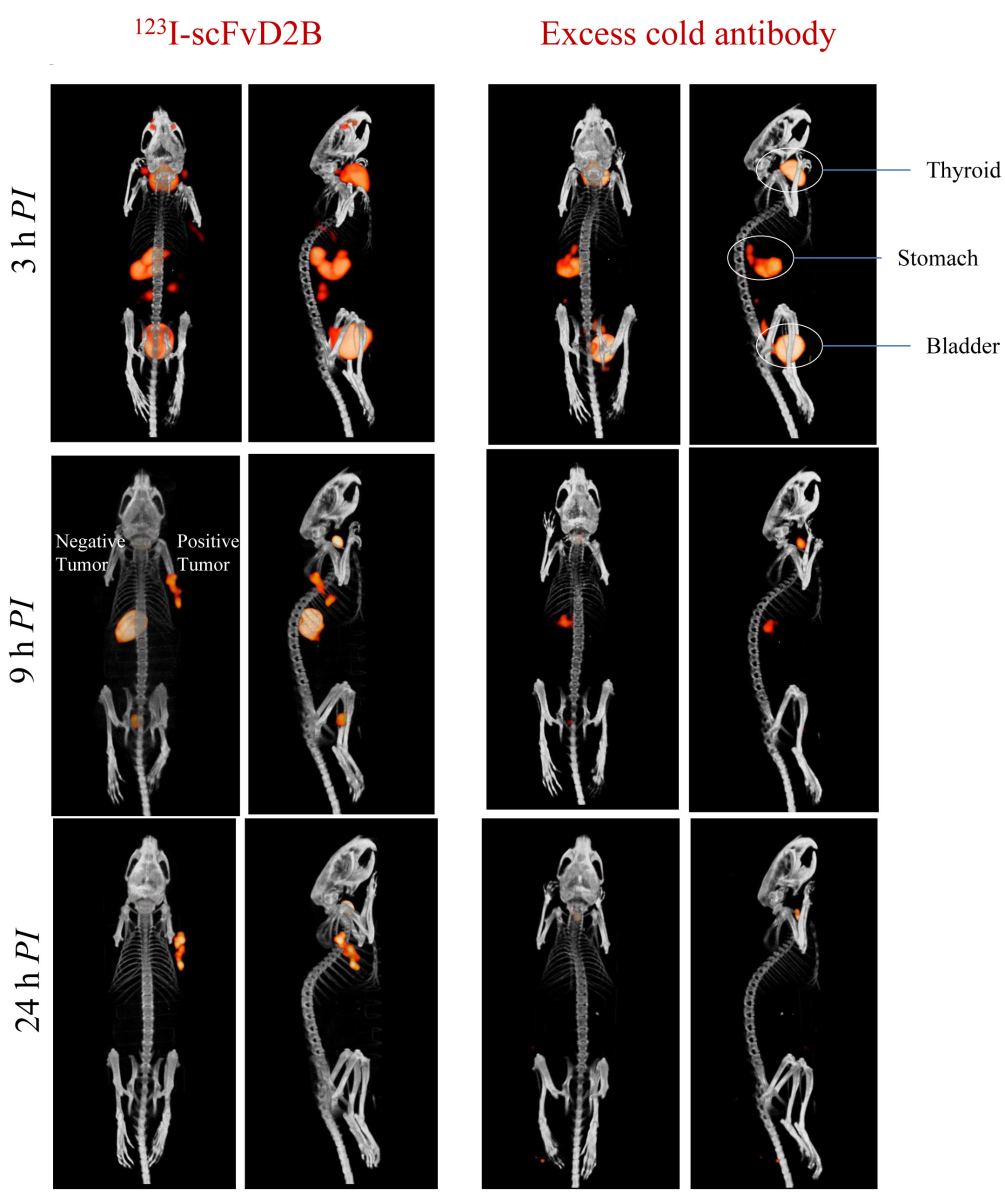
Representative SPECT/CT images in LS174T-PSMA/LS174T model after intravenous administration of 123I-scFvD2B. PSMA-positive tumor LS174T-PSMA (right flank) and PSMA-negative tumor LS174T (left flank) evaluated at 3, 9 and 24 hours post injection. Left: 12 MBq (8 µg; SA = 1500 MBq/mg) administered. Right: 12 MBq administered plus a 100-fold excess of cold scFvD2B.

MicroSPECT imaging data after intravenous injection of the ^123^I-scFvD2B fragment demonstrated significant and specific uptake in PSMA-positive tumors (LS174T-PSMA), while the signal measured in PSMA-negative tumors (LS174T) was similar to the signal in the non-target organs (Figure 4). The tumor-specific signal was already clearly detectable in PSMA-positive tumors nine hours after injection. To further test the specificity of the reagent, a group of animals was coinjected with a 100-fold excess of non-labeled antibody, resulting in almost undetectable tumor localization (Figure 4).

SPECT/CT performed using the PC3-PIP/PC3 model confirmed the specific tumor uptake in a mouse bearing a PSMA-positive tumor, while no signal was observed in the PSMA-negative tumor or in other organs (Figure 3 Supplementary).

## DISCUSSION

The aims of this study were the optimization of the anti-PSMA antibody fragment scFvD2B, the optimization of the radiolabeling procedure with ^123^I, and the validation of ^123^I-scFvD2B in preclinical models in order to bring it to the clinic for PCa imaging. We previously produced scFvD2B in a prokaryotic system and tested that reagent radiolabeled with ^131^I and ^111^In in preclinical *in vivo* models. ^131^I-scFvD2B yielded a significantly better target/background ratio than ^111^In-scFvD2B, mainly due to a remarkable reduction in kidney uptake [16].

In the roadmap for a useful clinical-grade reagent, our first step was to change the production system of the antibody fragment. In GMP eukaryotic production, removal of the His and Myc tags present in the reagent produced in a prokaryotic system, was dictated by the need to reduce the immunogenicity and to adhere to the directions of the regulatory agency. Whereas no change in affinity was seen in the two products, this modification resulted in a MW decrease and a pI increase of the eukaryotic reagent. It has been reported that modification of these parameters may change the biodistribution and targeting properties of radiolabeled reagents, with a reduction of kidney and liver accumulation as described by Hofstroöm et al [17].

The second step was to replace ^131^I with ^123^I; in fact, the physical properties of ^123^I (half-life 13 hours compared with 8,021 days, gamma emission of 159 keV compared to 971 keV, lack of beta emission) are highly favourable for diagnostic purposes, with an expected higher target/background ratio and reduced radiation exposure for healthcare professionals. Our present findings do not allow us to ascertain which of these modifications drove the improvement in the immunoreactivity of eukaryotic ^123^I-scFvD2B with respect to the prokaryotic product, because we did not evaluate how single parameters influenced the improvement of the reagent. However, we minimized the uptake in normal tissues and improved the uptake in antigen-positive tumors. Moreover, the radiolabeling procedures were modified by introducing ascorbic acid instead of BSA and a sodium phosphate buffer instead of TRIS (which is not accepted by the regulatory agency for use in humans) without any loss of reactivity. Even a 10- or 50-fold increase in specific activity did not reduce the immunoreactivity of the reagent.

As the final step we assessed the biodistribution and imaging of ^123^I-scFvD2B in three preclinical *in vivo* models and two different laboratories. Comparison of blood and tumor clearance of ^123^I-scFvD2B with data obtained previously with ^131^I-scFvD2B [16] suggested an improvement due to a clear trend toward a shorter circulatory half-life and a longer retention in PSMA-positive tumors. The first model to confirm the ability to target PSMA was the LNCaP cell line, which naturally expresses the target antigen of interest. To demonstrate the specificity we also used PC3-PIP cells, i.e., PCa tumor cells transfected with PSMA, in parallel with isogenic original PSMA-negative PC3 cells. A third preclinical model expressing very low PSMA levels (LS174T-PSMA) with respect to the other two models was analyzed in a different laboratory under different experimental conditions. Despite these differences, comparable highly specific localization was obtained in all models and the biodistribution in normal organs was characterized by low background in kidneys, liver and spleen. Additionally, in the LS174T-PSMA model the specificity was further demonstrated using both the isogenic original PSMA-positive cell line and excess cold antibody.

In the context of clinical translation, clear tumor visualization with ^123^I-scFvD2B at a time point, around of six-eight hours post injection, provides the basis for easier management of PCa patients. In fact, clinical imaging on the day of injection *versus* the need for a second visit to the clinic three to six days after injection, as required with a ProstaScint^®^ exam, makes it possible to avoid extended radioexposure to both patients and bystanders. Imaging acquisition at earlier time points are impaired due to high blood levels of the reagent.

It is known that an antibody molecule, in the intact format, have a long life *in vivo*, for IgG antibody the plasma half-life is 2-3 days; on the contrary, a scFv being an antibody fragment (6 time smaller than an entire antibody) is rapidly cleared from blood circulation. In fact, as demonstrated in Figure 2, no ^123^I-scFvD2B was detected in the blood after 15 hours allowing us to reduce the time of diagnosis as compared with the only FDA approved antibody Prostascint^®^.

In conclusion, since the current guidelines for patients with PCa indicate that they may be managed with different treatments ranging from active surveillance to external-beam radiation or brachytherapy to radical prostatectomy, a powerful reagent such as ^123^I-scFvD2B could represent a useful molecular imaging option to characterize and stage primary PCa, monitor disease progression, and assess the response to treatment; all these evaluations would ultimately lead to better management of PCa patients. In addition PSMA expression is dramatically up-regulated in poorly differentiated, metastatic and hormone-refractory carcinomas, as well as after androgen deprivation therapy and in lymph node metastases and for this reason we assume that the uptake in this cohort of patients could be optimal [18].

Moreover, given the encouraging results of new PCa target therapies that are based on PSMA recognition, i.e., ADC therapies [19] or immunological therapies [20], the availability of PSMA-tracking reagents that act as predictive biomarkers for patient selection and management helping to assess treatment response and biochemical recurrence, could be of interest.

## MATERIALS AND METHODS

### Characterization of scFvD2B from eukaryotic production

The scFvD2B used in this study was produced in GMP-compliant conditions in a eukaryotic system (ExcellGene) starting from a clone in which the Myc tag and His tag were removed. The antibody fragment was purified using a HiTrap Protein L chromatography column (GE Healthcare Life Sciences) according to the manufacturer's instructions and characterized for integrity and biochemical and biological activity as described elsewhere [13, 16].

The theoretical and experimental molecular weight (MW) and isoelectric point (pI) of scFvD2B produced in a prokaryotic system and an eukaryotic system were determined. The theoretical values were obtained using the ExPASy tool (http://web.expasy.org/compute_pi/); experimental MW and pI were calculated by SELDI-TOF (Bio-Rad laboratories) analysis and isoelectrofocusing gel (pH 3-9) using the PhastSystem (GE Healthcare Life Sciences), respectively.

### Cell lines

The PC3, LNCaP (human prostate cancer), LS174T (colon carcinoma) and A431 (vulvar epidermoid carcinoma) cell lines were purchased from the American Type Culture Collection (ATCC). The PC3-PIP cell line, which is isogenic to PC3 and stably expresses PSMA [21], was kindly provided by Dr W. Heston (Cleveland, OH). LS174T cells were transfected with human PSMA as described [22] and the resulting LS174T-PSMA clone stably expressed PSMA. Cell lines were subjected to short tandem repeat analysis in accordance with the ATCC guidelines, and the genetic profiles were compared with those of publicly available databases to verify their authenticity. Cells were maintained *in vitro* at 37°C in a humidified atmosphere of 5% CO_2_/95% air as follows: LS174T-PSMA cells were maintained in RPMI 1640 and 10% fetal bovine serum (FBS) in the presence of 0.3 mg/mL G418; LS174T cells were maintained in Eagle's minimal essential medium and 10% FBS; the other cell lines were maintained in RPMI 1640 and 10% FBS.

### Flow cytometric analysis

For cell surface staining, LNCaP, PC3-PIP, LS174T-PSMA and, as negative controls, PC3, A431 and LS174T cells were incubated with 10 µg/mL scFvD2B in phosphate-buffered saline (PBS) with 0.03% bovine serum albumin (BSA) for one hour on ice. After incubation, samples were washed three times in PBS and incubated for one hour with 5 µg/mL of biotinylated Protein L (Sigma) on ice. Cells were washed three times in PBS and reacted with Streptavidin-PE (1:400, Invitrogen) for staining. They were then run through a Fortessa flow cytometer (BD Biosciences) and analyzed using the FlowJo software package.

### BIAcore analysis

The binding of scFvD2B obtained in a eukaryotic or prokaryotic system was evaluated at 25 °C by surface plasmon resonance using a Biacore 2000 instrument equipped with research-grade CM5 sensor chips (Biacore AB). The antibody 7E11, which recognizes the cytoplasmic tail of PSMA, was immobilized on CM5 sensor chips as described previously [23]. LNCaP and PC3-PIP lysates (0.5 mg/mL) were injected three times at a flow rate of 30 µL/min for 10 minutes in order to enable the capture of soluble PSMA present in the lysate by 7E11 immobilized on the sensor chip. Both ScFvD2B were injected at 400nM at a flow rate of 30 µL/min for 3 minutes. The data obtained were analyzed with the BIAevaluation software 3.2.

### ^123^I-scFvD2B radiolabeling

Radioiodination of scFvD2B with ^123^I was performed according to the procedures of the two independent laboratories in Milan and Nijmegen. For the LNCaP and PC3-PIP models, Na ^123^I (GE Healthcare), along with cold NaI as carrier, was oxidized in an iodogen-coated tube (Pierce) with 0.1 mL of iodination buffer (25 mM TRIS-HCl, pH 7.4 + 0.4 M N aCl or PBS) for 5 minutes at room temperature and then added to scFvD2B for 10 minutes at room temperature (range 90-190 MBq ^123^I per mg of scFvD2B). The radiolabeled reagent was purified using a PD-10 desalting column and eluted with 100 mM sodium phosphate buffer, pH 7.4, and 150 mM NaCl containing ascorbic acid 10 mg/mL. The fractions corresponding to the radioactive peaks were pooled and counted in a Packard Cobra II automatic gamma counter. The labeling efficiency and the radiochemical purity, calculated as the amount of radioactivity associated with the protein *versus* the free fraction available after the reaction, were determined by instant thin-layer chromatography on silica gel strips (ITLC-SG; Gelman Sciences) using a methanol/physiologic sodium chloride solution (1:1, v/v) as the mobile phase, read by a Cyclone Storage Phosphor System (PerkinElmer), and analyzed by the Optiquant image analysis software (Packard).

For the LS174T model, scFvD2B was radioiodinated with ^123^I as described above with minor modifications: 93 MBq Na ^123^I, along with cold NaI as carrier, was added to a vial coated with 100 μg iodogen in 0.1 mL of 0.1 M phosphate buffer, pH 7.4. After 5 minutes at room temperature, 40 μg scFvD2B was added and incubated for 10 minutes at room temperature. The labeling efficiency was determined using ITLC-SG (Agilent Technologies) with 0.1 M citrate buffer (Sigma-Aldrich), pH 6.0, as the mobile phase. The reaction mixture was purified on a PD-10 column and eluted with PBS containing 0.5% BSA (Sigma-Aldrich).

### Immunoreactivity

The specific immunoreactivity of ^123^I-scFvD2B was evaluated using 1×10^7^ freshly detached cells incubated with trace amounts of radiolabeled scFvD2B in 250 µL of 0.03% BSA in PBS for three hours at room temperature with gentle rotation. Then the cells were washed three times with cold buffer. The activity in the supernatants and in the cell pellets was determined in the gamma counter. The immunoreactivity was calculated as follows: (cell pellet radioactivity/cell pellet and unbound radioactivity) ×100.

### Preclinical animal models

All protocols were approved by the Ethics Committee for Animal Experimentation of the Fondazione IRCCS Istituto Nazionale dei Tumori (INT) or the Dutch Act on Animal Experiments (WOD) and performed according to institutional guidelines and the “Guidelines for the Welfare and Use of Animals in Cancer Research” [24]. Procedures involving animals and animal care were in conformity with INT institutional guidelines that comply with national and international laws and policies (D.L. 116/92 and subsequent implementing circulars).

Three different animal models were obtained by subcutaneous injection of the following cell lines in the flank of athymic mice: A) LNCaP, a human PCa cell line that naturally expresses PSMA; B) PC3-PIP (PSMA positive) and PC3 (PSMA negative), a human PCa bone metastasis cell line; C) LS174T-PSMA (PSMA positive) and LS174T (PSMA negative), a colon carcinoma cell line.

For the LNCaP and PC3 models, male CD1 athymic mice (6-7 weeks old) were purchased from Charles River Laboratories and acclimatized for at least one week before tumor cell injection. One volume (50 µL) of saline containing 5×10^6^ cells (LNCaP, PC3-PIP or PC3) was mixed with two volumes of Matrigel (BD Biosciences) and injected subcutaneously into the animals’ flanks.

For the LS174T models, male athymic mice (8-weeks old) were purchased from Janvier Labs and after acclimatization were subcutaneously injected with 1×10^6^ LS174T cells in the left flank and 2×10^6^ LS174T-PSMA cells in the right flank.

In each model, tumor growth was monitored over time and when the tumors reached an appropriate volume (200-400 mm^3^), non-tumor-bearing mice or mice bearing tumors in one or both flanks were injected *via* the tail vein with ^123^I-scFvD2B. In the LS174T models, a group of animals was coinjected with a 100-fold excess of cold scFvD2B.

### Clearance from blood and tumor and *ex vivo* biodistribution

Blood was collected at different time points after ^123^I-scFvD2B injection. At the end of the experiment the animals were euthanized and tissues of interest were dissected. Blood and tissue samples were wet-weighed and counted in a gamma counter with internal standard to correct decay. Measurements were expressed as percentage of the injected dose per gram of tissue (%ID/g).

### SPECT/CT

Animals from the PC3-PIP/PC3 model were injected intravenously with 7.4 MBq ^123^I-scFvD2B. Twenty-four hours after injection mice were anesthetized with ketamine/xylazine (80/10 mg/kg) and subjected to SPECT/CT (Symbia Intevo T6 system, Siemens; acquisition time 30 min).

Animals from the LS174T-PSMA/LS174T model were injected intravenously with 12.0 MBq ^123^I-scFvD2B per mouse. At three, nine and 24 hours after injection mice were anesthetized with isoflurane/O_2_ and images were acquired using a U-SPECT II microSPECT/CT scanner (MILabs) with a 1.0-mm diameter multi-pinhole collimator tube (acquisition time 20-90 min).

### Statistical analysis

Statistical analyses were performed with GraphPad Prism, version 5.02. Immunoreactivity was analyzed by two-tailed *t*-test; a *P* value < 0.05 was considered significant. The blood and tumor clearance curves were defined using one-phase decay function to fit the activity measurement in the blood or tumor(s) and to calculate the blood terminal half-life (t_1/2_ β) and its 95% confidence intervals (CI); the goodness of fit was defined by R square. The results of *in vivo* biodistribution were expressed as mean ± standard deviation (SD).

## SUPPLEMENTARY MATERIALS FIGURES AND TABLES





## Abbreviations

PSMA: Prostate Specific Membrane Antigen
scFv: single chain fragment variable
PCa: Prostate Cancer
SPECT/CT: Single photon emission computed tomography
PET: positron emission tomography
CT: computed tomography
DTPA: diethylenetriaminepentaacetic acid
IgG: Immunoglobuline
GMP: good manufacturing practices
MW: molecular weight
pIs: isoelectric points
FACS: Fluorescence-activated cell sorting
ID: injected dose
SELDI-TOF: Surface-enhanced laser desorption/ionization with time of flight
ITLC-SG: Instant Thin Layer Chromatography Medium with a silica gel
