# Supplementary material for: Full preclinical validation of the 123I-labeled anti-PSMA antibody fragment ScFvD2B for prostate cancer imaging
Source: Oncotarget. 2016 Dec 30;8(7):10919–30. doi: 10.18632/oncotarget.14229 (PMC5355234; doi:10.18632/oncotarget.14229)
Supplement: Supplementary file 1 [file oncotarget-08-10919-s001.pdf]

Full preclinical validation of the ^{123}I-labeled anti-PSMA antibody fragment ScFvD2B for prostate cancer imaging

Supplementary Material

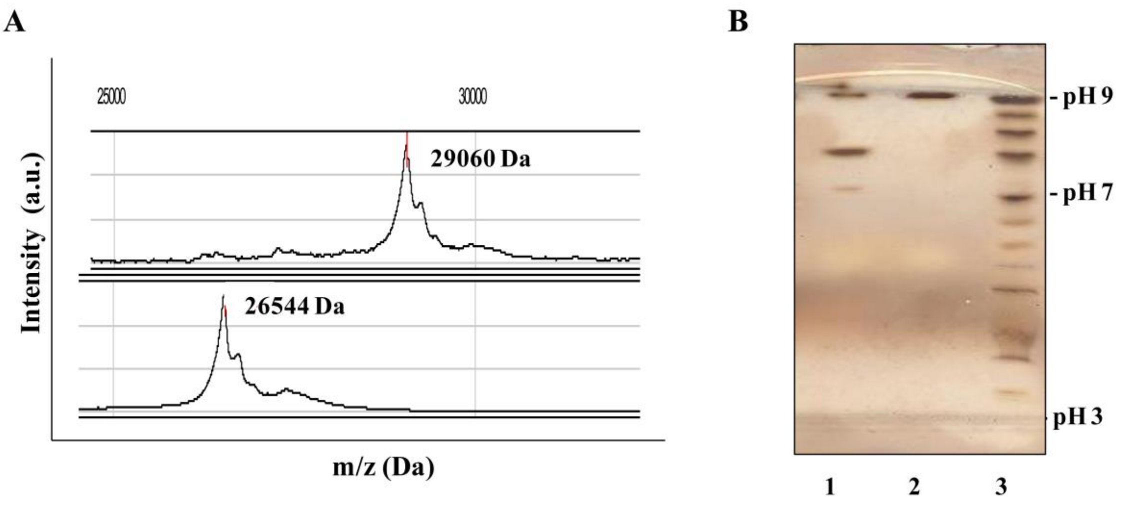

Supplementary Figure 1. Comparison of biochemical characteristics of scFvD2B.

A) SELDI-TOF analysis of scFvD2B from prokaryotic (upper peaks) and eukaryotic (lower peaks) system. **B)** Isoelectrofocusing analysis (gel 3-9) of scFvD2B from prokaryotic (lane 1) and eukaryotic (lane 2) system; markers (lane 3).

Removal of the His and Myc tags from the eukaryotic preparation resulted in a reduction of MW and increase in pI.

LS174T-PSMA/LS174T model

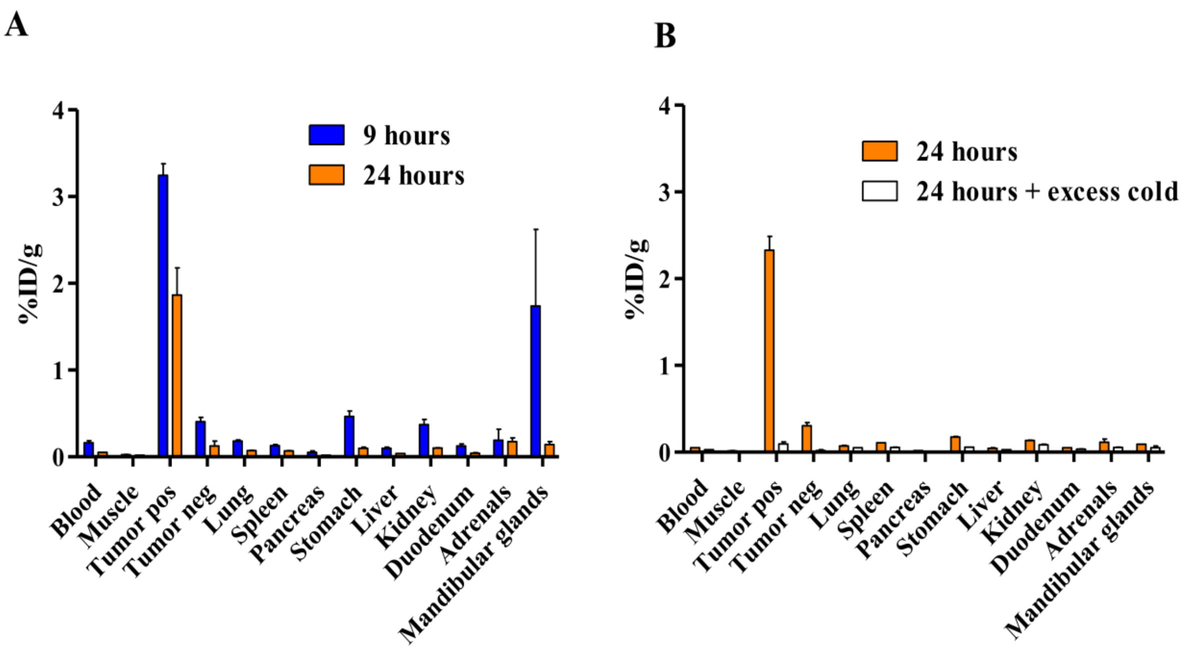

Supplementary Figure 2. Biodistribution and localization after intravenous administration of ^{123}I-scFvD2B in athymic mice. Uptake and retention measured in different organs as decay-adjusted percentage of injected dose per gram of tissue (%ID/g). **A)** Biodistribution in LS174T-PSMA/LS174T model evaluated at 9 and 24 hours post injection; 0.65 MBq (0.4 µg; SA = 1500 MBq/mg) administered; error bars represent SD from the mean value of 3 mice. **B)** Biodistribution in LS174T-PSMA/LS174T model evaluated at 24 hours post injection; 12 MBq (8 µg; SA = 1500 MBq/mg) administered with or without 100-fold excess of cold scFvD2B; error bars represent the SD from the mean value of 2 mice. The same animals were evaluated by SPECT/CT.

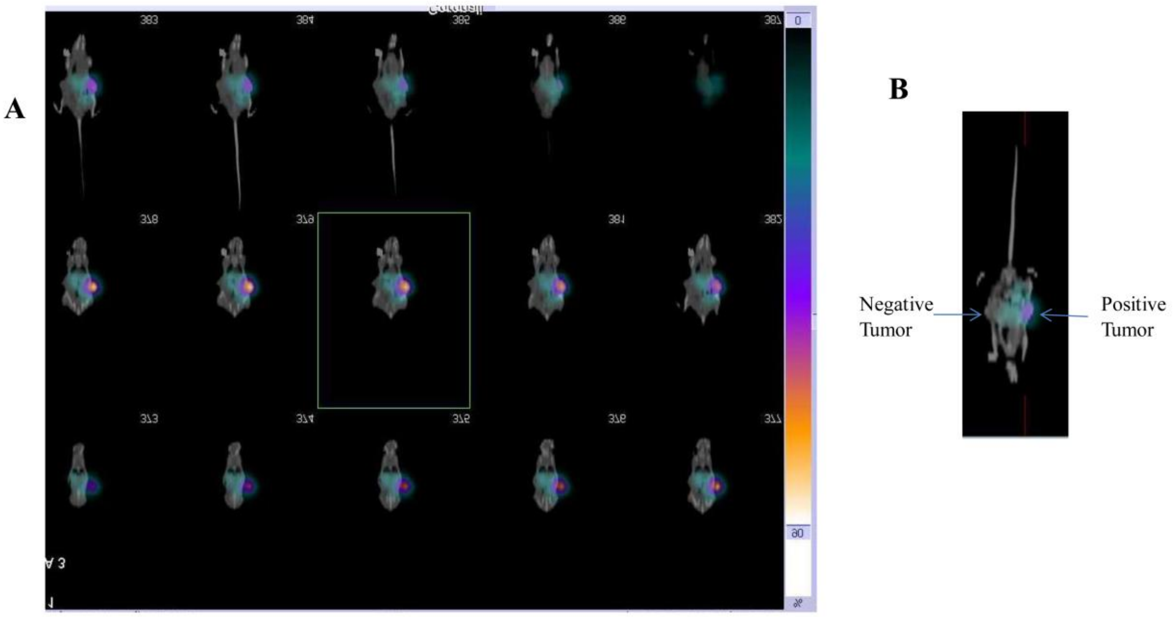

Supplementary Figure 3. SPECT/CT imaging. **A)** Representative SPECT/CT imaging performed 24 hours after injection of 7.4 MBq of ^{123}I-scFvD2B (51 μg; SA = 145 MBq/mg) . Coronal sections demonstrate high uptake of ^{123}I-scFvD2B in athymic mouse bearing subcutaneous PC3-PIP tumor while no uptake is evident in PC3 tumor or in other organs. **B)** Magnification of a section.
